# Determinants of intended prevention behaviour against mosquitoes and mosquito-borne viruses in the Netherlands and Spain using the MosquitoWise survey: cross-sectional study

**DOI:** 10.1186/s12889-024-19293-0

**Published:** 2024-07-04

**Authors:** Pauline A. de Best, Ayat Abourashed, Laura Doornekamp, Eric C. M. van Gorp, Aura Timen, Reina S. Sikkema, Frederic Bartumeus, John R. B. Palmer, Marion P. G. Koopmans

**Affiliations:** 1 Viroscience, Erasmus University Medical Center, Rotterdam, 3015 GD the Netherlands; 2 National Institute for Public Health and the Environment (RIVM), Bilthoven, 3721 MA the Netherlands; 3 Centre d’Estudis Avançats de Blanes (CEAB-CSIC), Blanes, 17300 Spain; 4 Department of Medical Microbiology and Infectious Diseases, University Medical Center, Rotterdam, 3015 GD the Netherlands; 5 Department of Primary and Community Care, RadboudUMC, Nijmegen, 6525 GA the Netherlands; 6 Athena Institute, VU University, Amsterdam, 1081 HV the Netherlands; 7 Centre de Recerca Ecològica I Aplicacions Forestals (CREAF), Cerdanyola del Vallès, Barcelona, 08193 Spain; 8 Institució Catalana de Recerca i Estudis Avançats (ICREA), Barcelona, 08010 Spain; 9 Department of Political and Social Sciences, Universitat Pompeu Fabra, Barcelona, 08005 Spain

**Keywords:** Surveys and Questionnaires, Vector-Borne Diseases, Mosquito-borne viruses, Health Belief Model, Knowledge, Social Determinants of Health, Prevention and control, Confidence Interval Based Estimation of Relevance Analysis

## Abstract

**Background:**

Recently, Europe has seen an emergence of mosquito-borne viruses (MBVs). Understanding citizens’ perceptions of and behaviours towards mosquitoes and MBVs is crucial to reduce disease risk. We investigated and compared perceptions, knowledge, and determinants of citizens’ behavioural intentions related to mosquitoes and MBVs in the Netherlands and Spain, to help improve public health interventions.

**Methods:**

Using the validated MosquitoWise survey, data was collected through participant panels in Spain (*N* = 475) and the Netherlands (*N* = 438). Health Belief Model scores measuring behavioural intent, knowledge, and information scores were calculated. Confidence Interval-Based Estimation of Relevance was used, together with potential for change indexes, to identify promising determinants for improving prevention measure use.

**Results:**

Spanish participants’ responses showed slightly higher intent to use prevention measures compared to those of Dutch participants (29.1 and 28.2, respectively, p 0.03). Most participants in Spain (92.2%) and the Netherlands (91.8%) indicated they used at least one prevention measure, but differences were observed in which types they used. More Spanish participants indicated to have received information on mosquitoes and MBVs compared to Dutch participants. Spanish participants preferred health professional information sources, while Dutch participants favoured government websites. Determinants for intent to use prevention measures included “Knowledge”, “Reminders to Use Prevention Measures”, and “Information” in the Netherlands and Spain. Determinants for repellent use included “Perceived Benefits” and “Cues to Action”, with “Perceived Benefits” having a high potential for behavioural change in both countries. “Self-Efficacy” and “Knowledge” were determinants in both countries for breeding site removal.

**Conclusion:**

This study found differences in knowledge between the Netherlands and Spain but similarities in determinants for intent to use prevention measures, intent to use repellents and intent to remove mosquito breeding sites. Identified determinants can be the focus for future public health interventions to reduce MBV risks.

**Supplementary Information:**

The online version contains supplementary material available at 10.1186/s12889-024-19293-0.

## Background

Europe is increasingly confronted with mosquito-borne viruses (MBVs) such as chikungunya, West Nile virus (WNV), and dengue, resulting in autochthonous outbreaks of diseases these viruses cause [1–4].While these outbreaks have been limited in size, this may change with the expected impact of climate change and globalisation on MBV ecology [5].

The emergence of MBVs in Europe is, in part, a consequence of the expanding prevalence and geographical range of resident and invasive mosquito species. Local establishment of competent invasive vector species like *Aedes albopictu*s and *Aedes aegypti*, together with virus introduction, can result in local transmission of disease [6]*.* While the spread of *Aedes aegypti* in Europe is still limited to Madeira and Cyprus*, Aedes albopictus* is already an established species in most Southern European countries including Spain, Italy, and Greece. In addition, *Aedes albopictus* has been responsible for outbreaks of various MBVs [5, 7]. For example, recurring clusters of dengue have been reported in Southern Europe, including 72 autochthonous cases in mainland Italy in 2023 [4]. While Northern European countries are seeing an increase in *Aedes albopictus* introductions (including in the Netherlands and Germany), the species is not yet established in these countries [5]. However, locally established mosquito species like *Culex pipiens* also are competent vectors of WNV and other viruses [8]. Since its first emergence in Southern Europe, WNV has continued to spread with its most recent emergence in Germany in 2018 and the Netherlands in 2020 [1, 2].

Given these developments, preparing residents to prevent and understand disease risk for potential MBV outbreaks is increasingly important [5]. Human behaviour plays an important role in infectious disease control and prevention, although it is important to acknowledge that other factors such as socio-demographic variables often also play a crucial role in driving these efforts. This behaviour is shaped by many factors, also known as determinants, including attitudes, knowledge, and risk perceptions; however, it is important to acknowledge that socio-demographic variables can also play a crucial role in driving these efforts [9]. Understanding these determinants and related behaviours is considered important for designing effective communication and educational strategies for risk reduction [10, 11]. There is a body of literature measuring behavioural determinants related to mosquitoes and MBVs in (sub)tropical endemic regions, but far less for the European context. However, recently the MosquitoWise survey was developed and validated, focusing on residents in Europe [12].

Since risks and occurrence of MBV infections differ between Northern and Southern European countries, residents may have different perceptions and knowledge and, consequently, different behavioural determinants. For example, the Netherlands and Spain have had different experiences with WNV transmission recently. While autochthonous WNV cases have occurred in Spain since 2004, Spain experienced its most significant WNV outbreak in 2020, with 77 confirmed cases, ranking it second in Europe for the number of infections that season. However, only recently there has been local transmission of an MBV in the Netherlands, where the first eight autochthonous WNV cases occurred in 2020, and no new cases have been reported since [1]. In terms of *Aedes*-related viruses, no local cases of dengue have been reported in the Netherlands. This is in part due to the lack of *Aedes albopictus* establishment in the Netherlands [5]. Whereas, in Spain, *Aedes albopictus* mosquitoes have been expanding their geographical spread across the country since 2004, and 16 autochthonous cases of dengue have been reported between 2018 and 2023 [13, 14]. We hypothesize that Southern European and Northern countries will have different understandings of mosquitoes and MBVs based on differences in MBV exposure, and, thus, different prevention measure behaviours. Therefore, we aimed to assess and compare perceptions, knowledge, and behaviour towards mosquitoes and MBVs in a Southern European country (Spain) and a Northern European country (the Netherlands) using the MosquitoWise survey. Additionally, we analysed the data to identify and compare behavioural determinants to target in future public health interventions.

## Methods

### Study design and settings

This cross-sectional study used the validated MosquitoWise survey [12]. This survey is evaluated by experts and tested for validity and reliability using Confirmatory Factor Analysis and Cronbach’s alpha in representative groups of residents in the Netherlands and Spain. The MosquitoWise survey is based on the Health Belief model (HBM) [12], a well-known theoretical model that measures people's perceptions of health risks and other factors influencing their health behaviour. In conjunction with the HBM, this survey measures people's knowledge and perceptions and determinants of their behavioural intentions [12].

### Study population and data collection

We aimed to obtain a representative sample of the general population of the Netherlands and Spain by using a participant panel (Bilendi), which consists of individuals who have agreed to participate in research studies, often for a small incentive. Participant recruitment was age- and sex- stratified by the panel providers to match the general population of the Netherlands and Spain for inhabitants of 18 years and older. In addition to the age-and sex stratification, eligible participants were people 1) with residency in either the Netherlands or Spain and 2) that are at least 18 years old. Participants were excluded if they 1) did not complete the survey within the allotted time limit (2–25 min) or 2) did not complete the entire survey. 3) Two control questions (“Please select "Somewhat agree" as your answer choice” and “Please select "Disagree" as your answer choice”) were added to the survey as an additional check to see if participants completed the survey with authentic responses. If both control questions were answered incorrectly, participants were excluded [12, 15].

Data collection occurred from July 20th to September 30th, 2022 in three phases to distribute responses across the summer period [16]. Participants were directed from the panel environment to the online survey, where they were informed about the study aims, their right to withdraw from the study, and assured their data would be stored anonymously. During each phase, around 150 panellists were invited. Participants were compensated (0.67 euro value per 10-min survey), and responses were collected and stored in the online LimeSurvey platform.

### Survey and study measurements

MosquitoWise consists of 55 mandatory questions, including 19 validated HBM questions. According to the HBM, a person's beliefs related to health and the effectiveness of recommended health behaviours together predict the likelihood or intent of adopting preventive behaviours. This is measured using the following HBM constructs: Perceived Susceptibility (Susceptibility), Perceived Severity (Severity), Perceived Barriers (Barriers), Perceived Benefits (Benefits), Self-Efficacy, and Cues to Action [12, 17]. Perceived Susceptibility is a person's perception of the risk of acquiring a MBV in their country of residence. Perceived Severity is an individual’s perception of the severity of MBVs and the potential consequences of such a viral infection. Perceived Barriers refers to obstacles a person might see/experience that will prevent them from using prevention measures. Perceived Benefits assesses a person's perception of the effectiveness and advantages of using prevention measures. Self-Efficacy refers to an individual’s confidence in their ability to apply prevention measures. Finally, Cues to Action are triggers that influence a person’s decision to use prevention measures. The additional 36 questions were directed at identifying potential determinants that might influence behaviour. These included questions on demographic characteristics, exposure and awareness (e.g. mosquito nuisance and receiving information on MBVs), knowledge on mosquitoes and MBVs, prevention measure use, and perceived responsibility. Table 1 includes all questions mentioned in this manuscript.

**Table 1:** Overview of questions with corresponding question codes mentioned throughout the manuscript, tables, and figures

Question Code	Question	Answer Choices
KNbite	Mosquitoes only bite people during the day	Single Answer
KNbreed	In gardens, mosquitoes can lay eggs in:	Multiple Answers
KNvirus	Mosquitoes are the main spreaders of the following viruses:	Multiple Answers
KNroute	A person can possibly get a mosquito-borne virus if:	Multiple Answers
ConstantPMuse	I remember to apply prevention measures against mosquitoes during mosquito season (March to September)	7-point Likert scale
MBVworry	I am worried about getting sick from a mosquito-borne virus in my country of residence	7-point Likert scale
LongClothes	In hot weather, wearing long-sleeved shirts and long trousers as a prevention measure against mosquito bites is uncomfortable	7-point Likert scale
Information	I have you read or heard any information about mosquito-borne viruses	Multiple Answers
RESGovRemBreed	I think the government is mainly responsible for removing mosquito breeding sites in my neighbourhood	7-point Likert scale
RESSelfRemBreed	I think I am mainly responsible for removing mosquito breeding sites in and around my house	7-point Likert scale

*KN* Knowledge, *PM* Prevention Measure, *MBV* Mosquito-Borne Virus, *Gov* Government, *RES* Perceived Responsibility, *RemBreed* Breeding Site Removal, *7-point Likert scale* 1 Strongly Disagree, 2 Disagree, 3 Somewhat Disagree, 4 Neutral, 5 Somewhat Agree, 6 Agree, 7 Strongly Agree

The main outcome measures of the survey were the mean HBM construct scores (1–7 points possible) and the total HBM score (6–42 points possible). The total HBM score represents a participant’s overall intent of adopting preventive behaviours based on individual scores from various HBM constructs. In the context of the MosquitoWise survey, this overall score is defined as a participant's intent to use prevention measures against mosquitoes and MBVs. Each question response was scored on a seven-point Likert scale from “Strongly Disagree” (1) to “Strongly Agree” (7) [12]. The HBM construct scores were calculated by taking the mean of all questions within a construct. To calculate the intent to use prevention measures (HBM score), first, the responses to the “Barriers” questions were reversed scored, so a higher “Barriers” mean score indicated there are no barriers for prevention measure use and, thus, a higher intent to show preventive behaviour. Then, all construct scores were aggregated to create the final HBM score. A low overall HBM score reflected a low intent to show preventive behaviour, and a high score revealed a high intent to show preventive behaviour. Other main outcomes included participants' use of self-reported prevention measures, which were selected based on recommendations from public health authorities (refer to ‘Additional file 1’ for more details about each prevention measures). We also examined the reasons participants used these prevention measures. Additionally, “Knowledge” was assessed and grouped into two main categories: mosquito knowledge (0–3 points possible) and MBV knowledge (0–6 points possible). The number of correct answers was calculated, resulting in a total knowledge score from zero to nine points. Lastly, where people had received information on mosquitoes and MBVs and where they would prefer to find that information was also measured as they could be relevant determinants for behaviour and crucial insight for creating future communication campaigns. Based on these responses an information exposure score (called “Information”) was calculated. For each secondary information source (Social media, Family and friends, Television and news channels, Print newspapers, and Radio) selected, a participant received one point. A participant received two points for each primary information source selected: Health professionals, Government website, Educational institutes, Institutional websites, and Communication campaign. The points were then summed to create the information exposure score with a range of 0–15 points.

### Statistical analysis

Descriptive statistics were used to provide an overview of the Dutch and Spanish respondents which included demographic characteristics, knowledge levels, prevention measure use, HBM construct scores, and total HBM scores. Means, medians, frequencies, standard deviations, and interquartile ranges were calculated as appropriate to summarize the data.

The multi-group confirmatory factor analysis used to assess the survey’s comparability between countries is described in Additional file 2. Given the distribution of the data, differences between Dutch and Spanish respondents were assessed using non-parametric statistical tests. Specifically, chi-square tests and Fisher's exact tests were employed to evaluate differences in demographic characteristics and self-reported prevention measure use, while the Wilcoxon Rank Sum test was used to compare differences in knowledge scores, HBM construct scores, and total HBM scores between the two countries. Significance levels were assessed at *p* < 0.05.

### Confidence Interval Based Estimation of Relevance Analysis (CIBER)

Confidence Interval Based Estimation of Relevance Analysis (CIBER) is a method to identify the most relevant determinants for behaviour to target in behavioural change interventions. In our study, we chose CIBER analysis over traditional regression analysis. While regression analysis can identify the probability that determinants are strongly associated with a certain behaviour, it may overestimate the relevance of these determinants as intervention targets. This can occur if the distribution of determinant scores within the population are skewed and may only be relevant for a small segment of the population. CIBER analysis visualizes both the strength of association and the distribution of determinants within the population, allowing for a more nuanced understanding of which determinants are most relevant for intervention targeting [18].

We used CIBER to assess the relevance of several determinants for intent to use prevention measures (HBM scores) and binaryCIBER for the use of two specific prevention measures (skin repellent use and mosquito breeding site removal) with binary answers.

CIBER combines two types of analyses: 1) univariate distribution of the mean value of each determinant, using diamond shapes with 99.99% confidence intervals (CIs) (left panel in the figures shown below) and 2) the point estimate for the correlation of each determinant with the target variable/outcome behaviour with its 95% CI (right panel in the figures shown below). The univariate distribution of the mean shows how much potential room for improvement through intervention exists for each determinant. For example, if the mean is low, there is more room for improvement (improving participants’ scores to high). The point estimates for correlation and their confidence intervals show if there is an association between the determinant and the outcome behaviour (intent to use prevention measures). Combining these two helps identify determinants with room for improvement, associated with behaviour, and, thus, the most relevant variables to target in interventions. The confidence interval of explained variance (R^2^) of all the determinants included is provided at the top of each plot. Behaviour outcome variables with a binary response (for example using skin repellent with responses yes or no) are visualized using binaryCIBER. The binaryCIBER visualization distinguishes the univariate distribution of the means between participants who scored “no” and “yes” for “repellent use” with two diamond shapes (purple and green) with their 99.99% confidence intervals (left panel) and shows the bivariate associations (Cohen’s d) with 95% CI (right panel). In a binaryCIBER, the reported R^2^ in the plot is an indicator of the performance of the determinants as predictors for the binary outcome versus using no determinants to predict the outcome. Both Cox-Snell and Nagelkerke’s R^2^ (global R^2^) are reported [18].

Furthermore, we calculated the potential for change index (PCI). The PCI combines the univariate population distribution and the associations into an index that can be used to compare change potential of determinants. The PCI takes the product of the 1) difference between the determinant’s mean and the scale maximum and 2) the squared association with intention [19]. For CIBER, correlations were used as the measure of association. For binaryCIBER, Cohen’s d was used. A threshold of 0.90 or more was taken to indicate relevance of determinants. While the PCI serves as a convenient means to consolidate various information into a single quantitative metric, CIBER plots have the added value of showing the distribution of participants responses. Thus, we combined the PCIs and CIBER plots for our interpretation. CIBER and binaryCIBER plots and PCIs were created in R using the behaviorchange package [20, 21].

All analysis were performed using R version 4.3.0 [20].

## Results

### Participant characteristics

The survey was completed by 537 Dutch and 542 Spanish participants. After applying the exclusion criteria, 438 and 475 participants in the Netherlands and Spain, respectively, were included in the analysis. Participants in both countries were almost evenly distributed by binary self-reported gender (male and female). Participants were between 18 and 99 years old in the Netherlands and between 18 and 89 in Spain, and the median participant age was 49 in both countries. Most participants had at least post-secondary education and were employed (Table 2).

**Table 2:** Demographic characteristics of survey participants in the Netherlands and Spain, 2022

Survey Participant Characteristics		
**Characteristic**	**Netherlands**	**Spain**
	***N* = 438**	***N* = 475**
**Self-Reported Gender**	**Count (Percent)**	
Male	212 (48.04)	231 (48.63)
Female	225 (51.37)	242 (50.95)
Other	1 (0.23)	2 (0.42)
Prefer Not to Disclose	0 (0.00)	0 (0.00)
Age Group*		
18–29	109 (24.89)	82 (17.26)
30–39	62 (14.16)	76 (16.00)
40–49*	52 (11.87)	96 (20.21)
50–59*	58 (13.24)	100 (21.05)
60–69	102 (23.29)	83 (17.47)
70 ≤	55 (12.56)	38 (8.00)
**Education Level**		
Primary School	7 (1.60)	13 (2.74)
Secondary School	131 (29.91)	120 (25.26)
Post-Secondary School	295 (67.35)	339 (71.37)
Other	5 (1.14)	3 (0.63)
Occupation Status*		
Working*	225 (51.37)	290 (61.05)
Student	50 (11.42)	34 (7.16)
Homemaker	27 (6.16)	31 (6.53)
(Currently) Unemployed	33 (7.53)	42 (8.84)
Retired	103 (23.52)	78 (16.42)

For characteristics with significant differences in the chi-square testing, post-hoc analysis was performed

Fisher’s exact tests were used for variables including cells with less than five observations

^*^p value < 0.05. *p* values for pairwise comparison between the Netherlands and Spain were calculated using chi-square tests

### Intent to use prevention measures (HBM score) and knowledge

Differences were observed between participants in the Netherlands and Spain for all the mean construct scores and overall intent to use prevention measures (HBM score) (Table 3). In the Netherlands, participants had slightly higher mean scores for “Susceptibility”, “Barriers”, and “Self-Efficacy” than in Spain (*p* < 0.0001, *p* = 0.03, and *p* = 0.04, respectively). The overall mean HBM scores were 28.49 (Netherlands) and 29.05 (Spain) out of the maximum of 42 points. This suggests that, on average, participants in both countries have a moderate to moderately high level of intention to use prevention measures based on the HBM framework (Table 3).

**Table 3:** Overview of participants’ Health Belief Model scores, knowledge scores, and self-reported prevention measure use

Survey Participants Intent to Use Prevention Measures, Knowledge Scores, and Prevention Measure Use					
**Outcome Measure**	**Netherlands**		**Spain**		**Wilcoxon Rank Sum Test**
	***N* = 438**		***N* = 475**		
**Health Belief Model Scores**	**Mean (SD)**	**Median (IQR)**	**Mean (SD)**	**Median (IQR)**	***p* value**
Susceptibility	4.36 (0.99)	4.33 (1.33)	4.16 (1.21)	4.33 (1.67)	0.01
Severity	5.06 (1.17)	5 (2.00)	5.40 (1.05)	5.67 (1.33)	< 0.0001
Benefits	4.97 (1.04)	5 (1.33)	5.32 (0.90)	5.33 (1.33)	< 0.0001
Barriers	4.57 (1.29)	4.5 (1.50)	4.38 (1.31)	4.5 (2.00)	0.03
Cues to Action	4.98 (1.16)	5 (1.67)	5.39 (0.98)	5.33 (1.33)	< 0.0001
Self-Efficacy	4.57 (1.12)	4.5 (1.25)	4.40 (1.17)	4.5 (1.50)	0.04
Health Belief Model	28.49 (3.74)	28.54 (4.75)	29.05 (3.69)	29 (4.63)	0.03
**Knowledge Scores**	**Mean (SD)**	**Median (IQR)**	**Mean (SD)**	**Median (IQR)**	***p* value**
Mosquito Knowledge	2.46 (0.81)	3.00 (1.00)	2.37 (0.84)	3.00 (1.00)	0.11
MBV Knowledge	1.93 (1.59)	2.00 (2.00)	2.51 (1.61)	3.00 (3.00)	< 0.0001
Knowledge	4.39 (2.02)	4.00 (3.00)	4.88 (2.07)	5.00 (3.00)	< 0.0001
**Prevention Measure Use**	**Count (Percent)**				**chi-squared Test**
Long Sleeves/Pants	173 (43.03)		95 (21.69)		< 0.0001
Insect Repellent	238 (59.20)		267 (60.96)		0.57
Electric Zapper	158 (39.30)		66 (15.07)		< 0.0001
Electric Fan	109 (27.11)		95 (21.69)		0.08
Outlet Plug-in Repellent	112 (27.86)		226 (51.60)		< 0.0001
Window/Door Screens	243 (60.45)		209 (47.72)		0.0005
Bedroom Windows Closed	128 (31.84)		93 (21.23)		0.0007
Mosquito Bed Net	77 (19.15)		20 (4.57)		< 0.0001
Natural Methods	41 (10.20)		81 (18.49)		0.0006
Removing Breeding Sites	155 (38.56)		160 (37.53)		0.59
Other	9 (2.24)		15 (3.42)		0.30
None	36 (8.96)		37 (8.45)		0.81

*SD* Standard Deviation, *IQR* Interquartile range, *MBV* Mosquito-borne virus

*p* value < 0.05 is the threshold for statistical significance

For the Knowledge scores, the mean score for participants in Spain was higher than for those in the Netherlands (4.88 vs. 4.39, *p* = 0.00012, respectively) (Table 3). There was no difference in mosquito knowledge scores between the participants in each country (*p* = 0.11). However, participants in Spain had a higher mean score for the MBV knowledge questions than those in the Netherlands (*p* < 0.0001).

### Prevention measure use

Most participants in the Netherlands and Spain (91.04% and 91.55%, respectively) indicated they use at least one prevention measure (Table 3). Placing screens and applying repellent were among the most frequently used prevention measures for both countries. There were significant differences in the use of screens, long-sleeved shirts, and outlet plugins (Table 3). Removing breeding sites as a prevention measure did not differ between participants in each country. Most participants in both countries indicated they use prevention measures to reduce being bitten by mosquitoes. Using prevention measures to reduce the number of mosquitoes and the risk of being infected with an MBV was more frequently reported by participants in Spain than by those in the Netherlands. Of the 402 Dutch and 438 Spanish participants that reported using prevention measures, 83.58% of the Dutch and 80.82% of the Spanish respondents mentioned reducing mosquito biting as their main reason for prevention measure. Reducing the number of mosquitoes (51.24% for the Netherlands and 62.56% for Spain) and reducing the risk of MBVs (18.16% for the Netherlands and 28.08% for Spain) were less often selected as a reason for prevention measure use.

### Information sources and preferences

A clear difference was observed between the number of respondents receiving any information on mosquitos and MBVs in the Netherlands and in Spain (29.22% and 60.84%, respectively, *p* < 0.0001) (Additional file 3). For the “Information” score, the median scores were 0 points (Interquartile range = 1) in the Netherlands and 1 point (Interquartile range = 3) in Spain. In both countries, participants mostly received information through television and news channels (14.61% for the Netherlands and 36.63% for Spain). Furthermore, 18.38% of the participants in the Netherlands indicated they do not want to receive information compared to 6.26% of Spanish participants. Of the 370 Dutch participants who indicated they would like to receive information, they preferred Government websites (51.08%), Television and news channels (51.08%), Social media (38.92%), Radio (21.35%), and Communication campaigns (20.54%). In Spain, 447 participants indicated they would like to receive information and primarily from the following sources: Health professionals (60.63%), Television and news channels (59.06%), Communication campaigns (42.73%), Government websites (40.49%), and Institutional websites (36.24%) (Additional file 3).

### Confidence Interval-Based Estimation of Relevance (CIBER)

The complete questions with corresponding determinant codes in the CIBER and binaryCIBER figures can be found in Table 1.

### Behavioural determinants for intent to use prevention measures (HBM scores)

**Fig. 1:**
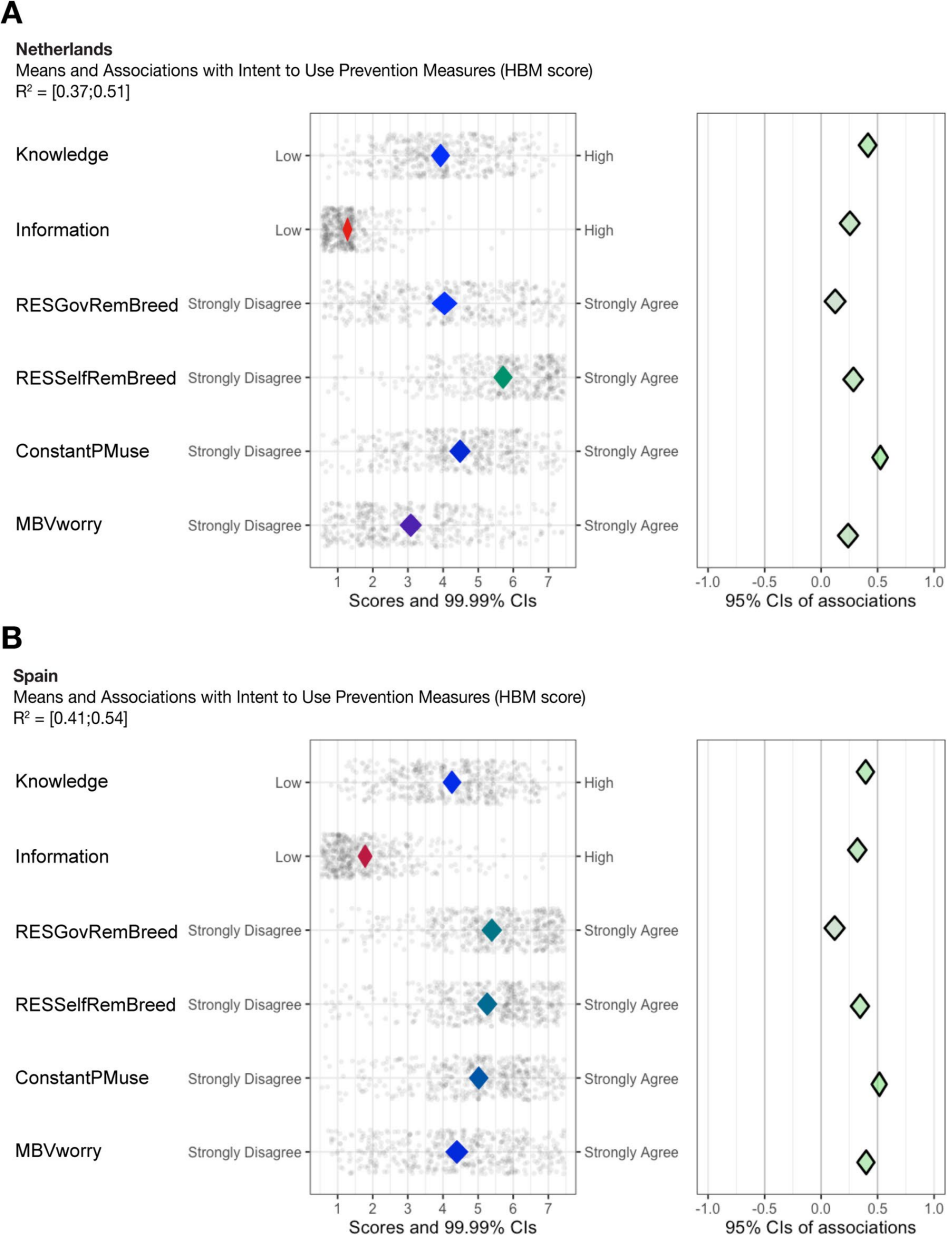
CIBER plots of the determinants for intent to use prevention measures (HBM scores) for **A** participants in the Netherlands and **B** those in Spain. In the leftmost panels, red diamonds indicate low means, green diamonds show high means, and blue diamonds illustrate middle means. The dots surrounding the diamonds in the left panel show participants’ response distribution with jitter to avoid overplotting. In the rightmost panels, the diamond’s colour demonstrates the strength and direction of association. Greener diamonds imply strong positive associations with intent to use interventions redder diamonds show strong negative associations, and greyer diamonds display weak associations. *HBM Score* Health Belief Model score. *CIs* Confidence Intervals. *R*^*2*^ Confidence interval of explained variance

Determinants for intent to use prevention measures (HBM scores) explained 37% to 51% of the variance among participants in the Netherlands and 41% to 54% of the variance for those in Spain (Fig. 1). For both countries, “Knowledge” and “ConstantPMuse” are two determinants that can serve as potential intervention targets because their mean scores are relatively closer to the middle and have positive associations with intent to use prevention measures. This indicates, that in both countries, participants with higher knowledge scores and participants who remember to apply prevention measures throughout mosquito season have a higher intent to use prevention measures.

The variables “Information” and “MBVworry” show a similar combination of relatively central means and positive associations; however, mean values were slightly higher for participants in Spain than for those in the Netherlands. This indicates that more participants in Spain read or heard information about MBVs compared to those in the Netherlands and that participants in Spain worry more about contracting a MBV in their country of residence than those in the Netherlands. “Information” and “MBVworry” have positive associations with HBM scores, which suggests that receiving information about mosquitoes and MBVs via multiple channels and worrying more about MBV risks are associated with a higher intent to use prevention measures. Question means were high for “RESSelfRemBreed”, indicating participants in both countries believe they are responsible for removing mosquito breeding sites in and around their houses. Figure 1 shows “RESSelfRemBreed” is positively associated with the intent to use prevention measures with above average mean responses.

In both the Netherlands and Spain, “Knowledge”, “Information”, “ConstantPMuse” and “MBVworry” all have PCIs above 0.90 (Table 4). This indicates these determinants are strong influencers for intent to use prevention measures in both countries. The PCIs also suggest that these four determinants have a higher potential to bring about change in people’s intent to use prevention measures. This implies that efforts focused on improving knowledge, providing information, enhancing personal confidence in prevention measure use and addressing perceptions of MBV risk could lead to meaningful changes in people’s intent to use prevention measures against mosquitoes and MBVs.

**Table 4:** Potential for Change for intent to use prevention measures, repellent use, and removing breeding sites in the Netherlands and Spain

Potential for Change							
**Outcome**	**Determinant**	**Netherlands**			**Spain**		
		**Mean Score**	**Weight**	**PCI**	**Mean Score**	**Weight**	**PCI**
**Intent to Use Prevention Measures**	Knowledge	3.93	0.42	**1.28**	4.26	0.40	**1.08**
	Information	1.28	0.26	**1.36**	1.78	0.32	**1.72**
	RESGovRemBreed	4.04	0.13	0.32	5.39	0.12	0.20
	RESSelfRemBreed	5.71	0.29	0.39	5.26	0.35	0.59
	ConstantPMuse	4.48	0.53	**1.25**	5.02	0.52	**1.00**
	MBVworry	3.08	0.24	**0.82**	4.40	0.40	**1.05**
**Repellent Use**	Susceptibility	4.36	-0.26	0.22	4.16	-0.13	0.05
	Severity	5.06	-0.27	0.31	5.40	-0.28	0.35
	Barriers	4.57	-0.21	0.15	4.38	-0.20	0.14
	Benefits	4.97	-0.69	**1.90**	5.32	-0.50	**1.10**
	Cues to Action	4.98	-0.54	**1.20**	5.39	-0.67	**2.00**
	Self-Efficacy	4.57	-0.15	0.08	4.40	-0.25	0.21
	Knowledge	3.93	-0.37	0.39	4.26	-0.29	0.28
	Information	1.28	-0.20	0.01	1.78	-0.39	0.12
	ConstantPMuse	4.48	-0.42	0.61	5.02	-0.56	**1.30**
	MBVworry	3.08	-0.10	0.02	4.40	-0.35	0.41
**Breeding Site Removal**	Susceptibility	4.36	-0.01	0.00	4.16	-0.12	0.05
	Severity	5.06	-0.18	0.13	5.40	-0.15	0.10
	Barriers	4.57	-0.32	0.36	4.38	-0.23	0.18
	Benefits	4.97	0.04	0.01	5.32	-0.11	0.05
	Cues to Action	4.98	-0.39	0.61	5.39	-0.39	0.68
	Self-Efficacy	4.57	-0.74	**1.90**	4.40	-0.53	**0.96**
	Knowledge	3.93	-0.54	0.84	4.26	-0.58	**1.10**
	Information	1.28	-0.12	0.00	1.78	-0.29	0.07
	ConstantPMuse	4.48	-0.35	0.43	5.02	-0.35	0.51
	MBVworry	3.08	-0.15	0.05	4.40	-0.01	0.00
	RESGovRemBreed	4.04	-0.12	0.04	5.39	-0.06	0.02
	RESSelfRemBreed	5.71	-0.61	**1.80**	5.26	-0.43	0.78

Values of the index larger or equal to 0.90 are shown in bold

*PCI* Potential for change index

### Behavioural determinants for repellent use

**Fig. 2:**
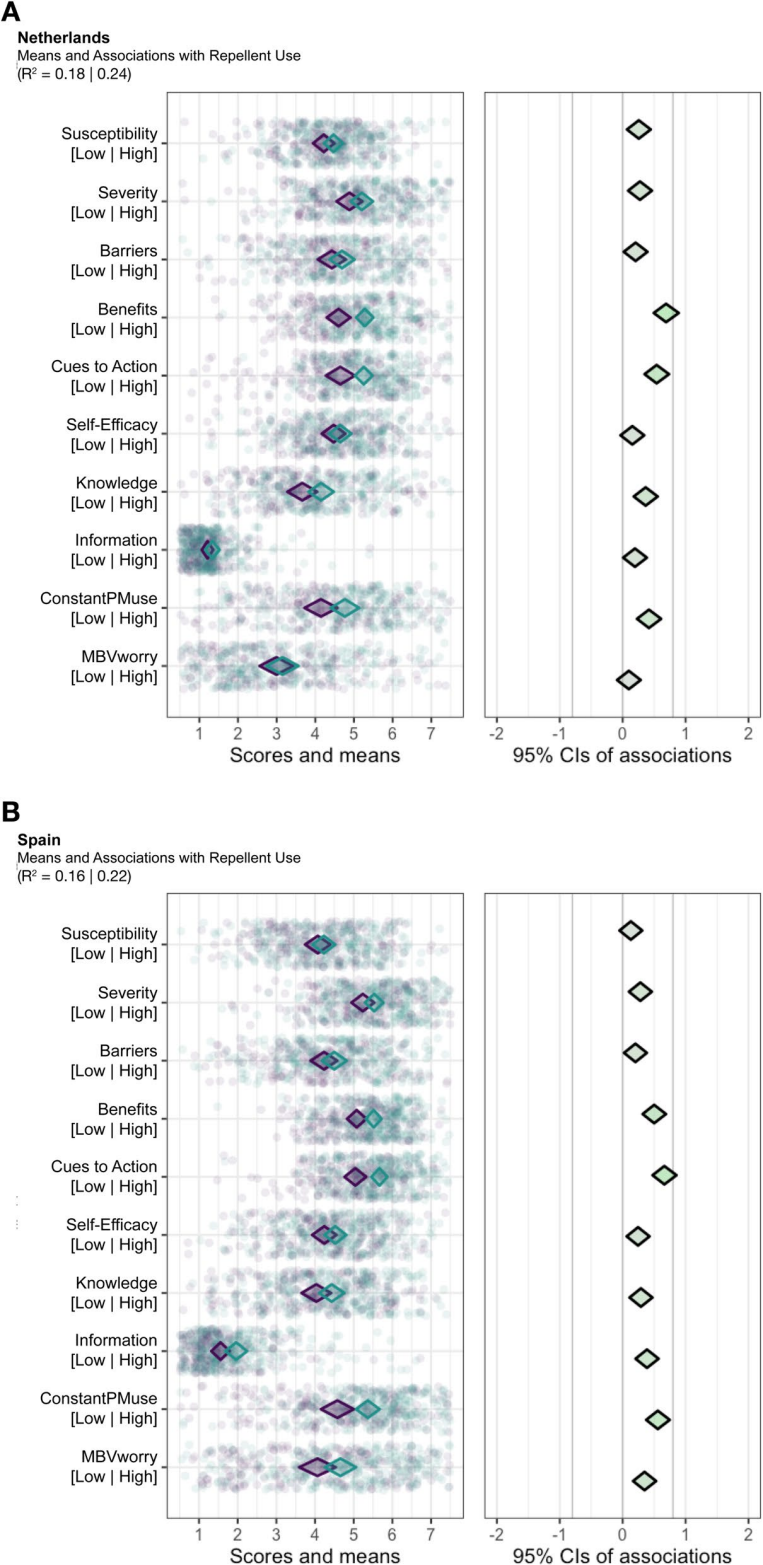
binaryCIBER plots of determinants for repellent use in **A** the Netherlands and **B** Spain. The leftmost panels have two diamonds: The teal diamonds are for participants that reported “yes” for using a specific prevention measure, and the purple diamonds are for those who said “no”. In the rightmost panels, the diamond’s colour demonstrates the strength and direction of association. Greener diamonds imply strong positive associations, redder diamonds show strong negative associations, and greyer diamonds display weak associations. *CIs* Confidence Intervals. *R*^*2*^ Cox-Snell | Nagelkerke

Associations between determinants and repellent use as a specific prevention measure are shown in Fig. 2. In both the Netherlands and Spain, similar determinants (“Benefits”, “Cues to Action”, and “ConstantPMuse”) are relevant for repellent use. This suggests that citizens see some advantages in using repellents as preventive actions against mosquitoes and MBVs and that being reminded to take preventive actions are potential reasons for using repellents. People’s confidence in using prevention measures can also be used as a concept for future interventions to improve citizens’ use of repellents. For both countries, "Knowledge" also had a slight positive association with roughly 50% of participants using repellents. Thus, improving knowledge can be a tool to improve repellent use.

PCI analysis for intent to use repellents, revealed “Benefits” and “Cues to Action” have a PCI above 0.90 for the Netherlands and Spain (Table 4). “ConstantPMuse” also has a PCI above the threshold but only for Spain.

### Behavioural determinants for breeding site removal

**Fig. 3:**
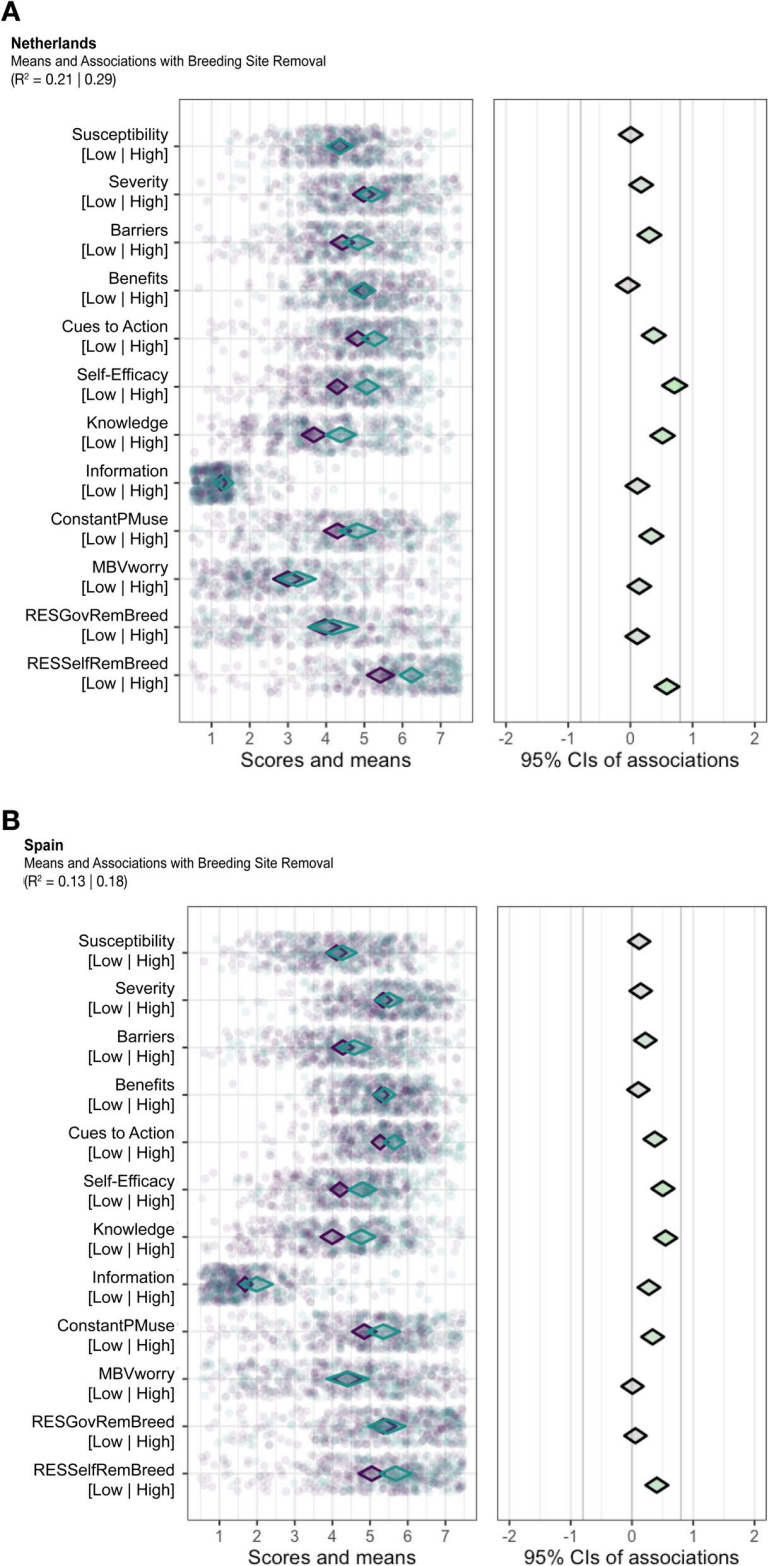
binaryCIBER plots of determinants for removing mosquito breeding sites in **A** the Netherlands and **B** Spain. The left panel has two diamonds: The teal diamonds are for participants that reported “yes” for using a specific prevention measure, and the purple diamonds are for those who said “no”. In the right panel, the diamond’s colour demonstrates the strength and direction of association. Greener diamonds imply strong positive associations, redder diamonds show strong negative associations, and greyer diamonds display weak associations. *CIs* Confidence Intervals. *R*^*2*^ Cox-Snell | Nagelkerke

Similar trends in determinant associations with breeding site removal are observed in both countries. “Self-Efficacy” and "Knowledge" have the strongest positive associations for participants removing breeding sites (Fig. 3). These determinants are important for future interventions because the mean scores are in the middle or higher for people who do remove mosquito breeding sites in and around their house in both the Netherlands and Spain. “RESSelfRemBreed” also has a positive association with breeding site removal; however, most participants in both countries have high means for this determinant.

The PCIs for breeding site removal determinants in participants in the Netherlands and Spain are different (Table 4). For the Netherlands, “Self-Efficacy” and “RESSelfRemBreed” have the greatest potential to influence behavioural change of breeding site removal. For Spain, "Self-Efficacy" and “Knowledge” have a PCI greater than 0.90.

## Discussion

Understanding people’s current knowledge, perceptions, and behaviours towards mosquitoes and MBVs is a crucial first step toward efficient information campaigns to involve citizens in the prevention of local epidemics of MBVs. The determinants for preventive behaviours identified in this study allow for informed recommendations for future communication campaigns for both the Netherlands and Spain. Furthermore, comparing knowledge, perceptions, and behaviours between participants from the Netherlands and Spain showed both differences and similarities. Assessing differences and similarities allows for a more robust understanding of public perceptions and offers the opportunity for context specific recommendations. Furthermore, using two countries from different European regions can provide insight in how regional variations in, for instance mosquito species and disease prevalence, may shape perceptions, knowledge, and behavioural intent, while enhancing the applicability of our findings to diverse European contexts.

### Intent to use prevention measures and reported prevention measure use

Since mosquito-borne viral disease incidence and prevalence and the presence of invasive mosquitoes such as *Aedes albopictus* are higher in Spain, we hypothesized that participants in Spain would have a higher perceived “Susceptibility” and “Severity” compared to those in the Netherlands. This is indeed the case for “Severity”, but not for “Susceptibility,” which was lower among participants in Spain. The “Susceptibility” construct combines being susceptible for mosquito bites and for contracting an MBV. Because of the presence of the day biting mosquito *Aedes albopictus,* a higher perceived susceptibility to bites was expected for participants in Spain [5]. However, their exposure also might lead to habituation to mosquito bites, especially considering the longer period for mosquito season in Southern European countries, as described for mosquito nuisance by Gaillard, et al. [22]. Our study is a stepping stone to understanding such relations between perceptions and behaviour, further context specific research could help to gain deeper understanding of the complexity of this relation. This study provides an overview of the situations of both countries and highlights some similarities and differences between the Netherlands and Spain.

Differences in intent to use prevention measures (HBM scores) were observed between the Netherlands and Spain. While these differences were small, they do indicate that the overall distributions of HBM scores of the two populations are different, which can be insightful when country specific communication campaigns are developed. However, the combined interpretation of CIBER plots and the PCIs showed that determinants influencing citizens’ intent to use prevention measures (HBM scores) are similar. Therefore, efforts to improve citizen’s overall intent to use prevention measures should focus on these main determinants: providing information, improving knowledge about mosquitoes and MBVs, and empowering citizens’ personal self-efficacy. A previous study in China showed that information about dengue had a direct positive association with people’s mosquito control behaviour [23]. Providing information is thought to enhance knowledge, which can ultimately increase people’s confidence in adopting preventive behaviours. In the study by Lun et al., providing information through mass media publicity (official WeChat accounts, magazines and newspapers, poster leaflets, television/radio, and the Internet) and organized publicity (medical staff and through community publicity) had both direct and indirect positive effects on dengue knowledge and mosquito control behaviour [23]. A study conducted in the United Kingdom showed that increased governance guidance on prevention measures against COVID-19 also increased protective behaviours [24]. Our study showed that participants would like to receive information via government websites and communication campaigns. Therefore, providing information and reminders to use prevention measures via these ‘government’ channels could improve intent to use prevention measures and, thus, actual prevention measure behaviour.

This study not only measured the intent to undertake preventive behaviour but also assessed reported prevention measure use and reasons for prevention measure use. While differences in the preference for certain prevention measures were observed between participants in each country, the main reason for prevention measure use was the same: to reduce being bitten by a mosquito. However, the presence of both day biting and night biting mosquito species in Spain could explain the preference of participants in that country for outlet plug-in repellents compared to those in the Netherlands. Plug-in repellents can typically release a steady stream of insect repellent, allowing continuous protection and a consistent barrier against day and night biting mosquitoes. In contrast, in the Netherlands, people are mostly at risk of being bitten during dusk and at night, and therefore might choose to use prevention measures only during that period. The use of bed nets, that was more common for participants in the Netherlands, also could be explained by a preference for using measures at night and dusk. These results may help explain current prevention measure practice and identify prevention measures that might need promotion in future communication campaigns. Nevertheless, more research in understanding why people use certain prevention measures and when they use them is crucial. Additionally, highlighting the advantage of using repellents to reduce biting nuisance and disease risk could be communication points for campaigns.

To improve the use of specific prevention measures, our results indicate more specific determinants should be targeted. To promote the use of mosquito repellents, highlighting the benefits, increasing knowledge and cues to action for repellent use, are concepts we recommend targeting. Educating people about the benefits of using prevention measures has been shown to be effective to enhance personal protective behaviours [25]. Therefore, similar effects are expected for repellent use if benefits are highlighted in future communication campaigns. Furthermore, Smith et al. showed that providing cues can improve preventive behaviours against disease risk [24]. Having consistent reminders for people to use repellents during mosquito season can complement these cues to action to potentially improve skin repellent usage.

To promote citizens removing mosquito breeding sites, our results indicate efforts should centre on enhancing citizen’s confidence in their breeding site removal skills ("Self-Efficacy"), knowledge acquisition about mosquitoes; their breeding sites; and MBVs; and informing people about how breeding site removal reduces mosquito presence in and around houses. The process of breeding site removal necessitates actionable efforts and knowledge from individuals. A comprehensive understanding of mosquito breeding habits, the ability to accurately identify potential breeding sites, and the continued execution of breeding site elimination throughout the mosquito season are essential to the process. Hence, it is not unexpected that these determinants are connected to breeding site removal. Previous studies on breeding site removal showed that knowledge on breeding sites alone is not sufficient to initiate behaviour as participants still lacked motivation [26, 27]. A previous study in Curacao revealed that higher perceived self-efficacy was positively associated with individuals removing mosquito breeding sites [27]. Therefore, combining knowledge on mosquito breeding habits with improving self-efficacy, could increase chances of adopting this behaviour.

### Knowledge

While the traditional HBM does not include knowledge as a construct, knowledge is considered a crucial component of people's perceptions and behaviours [28]. Participants in Spain had a slightly higher overall knowledge score than participants in the Netherlands. However, there was no difference in knowledge level regarding mosquito-related questions between the two countries. Mosquito populations exist in both countries, so residents are accustomed to dealing with mosquitoes. Furthermore, information campaigns, specifically related to *Aedes albopictus,* exist in both countries. In the Netherlands, national media reported on localized introductions of this invasive mosquito species and the approaches to prevent its establishment [29]. In Spain, *Aedes albopictus* has been established for years, especially along the Mediterranean coast, and media and citizen science initiatives request that people notify sightings of this mosquito [30]. However, participants in Spain did score significantly better on MBV-related questions, likely because MBV infections are more common in this region. The Netherlands had a few hospitalised human WNV cases due to local transmission in 2020, whereas, in Spain, human cases of WNV have occurred as early as 2004, and small viral outbreaks with viruses such as dengue and chikungunya, transmitted by *Aedes albopictus,* are reported yearly [1, 3, 4]. In the past decade, local municipalities in Spain have made greater and regular efforts to inform and educate citizens with campaigns at the start and during each mosquito season against *Aedes albopictus* and the disease threats it poses [31].This could be a potential explanation for the difference in MBV related knowledge between both countries. According to this study, knowledge has the potential to change people’s intent to use prevention measures and removing mosquito breeding sites. Although we observed differences in knowledge scores, the differences are small and enhancing knowledge is pertinent to improve intent to use and actual prevention measure use in both the Netherlands and Spain. Based on the survey results, we recommend television and news outlets as additional information outlets since they were preferred by participants in both countries.

### Limitations

This study does have some limitations. Most participants had at least secondary education and were employed. Our recruitment approach may have limited the diversity of the participant samples, potentially skewing results towards higher socio-economic groups and underrepresenting less educated or unemployed populations [12]. Using more targeted distribution strategies such as door-to-door surveys could be a means to reach these populations. Furthermore, our study focusses on the general public while large countries, like Spain, and even smaller countries, like the Netherlands, might experience regional differences in mosquito species’ distributions and disease prevalence. These regional differences might act as confounders and affect citizens perceptions and behaviour. Therefore, our current study provides valuable insights on a country level, but repeating this study in specific regions, with a range of environmental and socio-economic factors to consider, could offer more insights for targeted interventions. Additionally, participants were not asked about the frequency and consistency of their prevention measure use. Also, roughly 50% of intent to use prevention measures is explained by the determinants tested. This indicates there are other potential determinants that influence intention and confounding factors that this survey does not capture, such as ecological factors, socio-economic status of participants, and the amount of time participants spend outside in areas with mosquitoes [32–35]. Nonetheless, the information gained from this study is novel and helpful for future interventions, and longitudinal studies can be done using the same validated survey in the Netherlands and Spain.

### Public health implications and intervention recommendations

Our study findings can be applied to focus and improve the effectiveness of communication campaign messaging. For example, communication campaign interventions from multiple countries including the United States, Australia, the Netherlands, and Spain focus on encouraging citizens to apply simple prevention measures and, more specifically, to remove mosquito breeding sites [35–39]. Our findings suggest that participants encounter barriers that hinder their widespread adoption. Addressing these identified barriers and highlighting the benefits in communication campaigns can help convince people to improve prevention measure adoption.

Furthermore, the identified determinants show knowledge should be improved to increase intent to use prevention measures such as breeding site removal. By measuring knowledge for mosquitoes and MBVs in our study, we identified key knowledge gaps which can be used to improve communication campaigns. While the identified determinants were similar, we did observe differences in the secondary outcomes between the countries: reported prevention measure use, knowledge, and information exposure. These differences, even if small, do indicate that conducting surveys in specific countries can reveal specific intervention targets, such as MBV knowledge. Therefore, while our findings can enhance communication messages and are applicable to multiple European countries, we recommend conducting surveys in each specific country to tailor interventions to the local context.

Additionally, across Europe, climate change is bound to impact mosquito populations and, with that, the MBV transmission dynamics, making a tailored approach per country even more relevant. Warmer temperatures altered rainfall patterns, and increased humidity can create more suitable environments for mosquitoes, like *Aedes* mosquitoes, in more northern countries in Europe [2, 5]. This in turn can change mosquito seasonality and cause longer periods for mosquito activity and prospective overwintering of mosquitoes. These climatic changes leading to geographical expansion of invasive mosquito species can possibly introduce MBVs to these regions [2, 5, 6]. With climate change projecting to shift many influential factors, adapting public health strategies in anticipation of mosquito population dynamics and virus transmission potential is necessary more than ever. This underscores the importance of sustained public health campaigns that are adaptable to the shifting landscape of MBV threats. Therefore, we recommend periodical repetition of the MosquitoWise survey over the years to monitor intervention effectiveness after implementation by measuring behaviour change in the population. These periodic re-evaluations of campaigns are crucial to ensure their effectiveness and relevance especially amidst ongoing environmental changes.

Moreover, other distribution techniques can be considered for future distribution of the survey, including door-to-door, telephone, and online social media platforms. This might reach specific populations, like citizens living close to MBV cases or in neighbourhoods with low socio-economic status, that might benefit from more targeted communication efforts. Moreover, the MosquitoWise survey is already available in five languages (English, Dutch, Spanish, French, and Flemish) and can be translated to other languages to be distributed to more European countries, thus, allowing for standardized comparisons. Finally, repeating this study among (school) children, who are also at risk of MBVs, could help create targeted interventions for this audience and educate a new generation, although this would require validation of the survey for this specific target population [40].

## Conclusion

As climate changes and European countries become more suitable for new mosquito species and arbovirus transmission, understanding current beliefs and behaviours is a crucial first step to the design of prevention and control measures. While the situation and landscape in the Netherlands and Spain are different, determinants of intent to use prevention measures and to use prevention measures such as skin repellent and removing breeding sites are similar. With little literature on this topic for Europe, this study is a springboard to a more thorough understanding of how crucial behavioural sciences are for prevention and control interventions for infectious diseases.

### Supplementary Information

Additional file 1.

Additional file 2.

Additional file 3.

## Data Availability

The datasets used and analysed during the current study are available from the corresponding author on reasonable request.

## Abbreviations

MBVs: Mosquito-borne viruses
WNV: West Nile virus
Susceptibility: Perceived Susceptibility
Severity: Perceived Severity
Barriers: Perceived Barriers
Benefits: Perceived Benefits
CIBER: Confidence Interval Based Estimation of Relevance Analysis
Cis: Confidence intervals
PCI: Potential for change index
SD: Standard deviation
IQR: Interquartile range
KN: Knowledge
PM: Prevention measure
Gov: Government
RES: Perceived responsibility
RemBreed: Breeding site removal
