# Supplementary material for: Determinants of intended prevention behaviour against mosquitoes and mosquito-borne viruses in the Netherlands and Spain using the MosquitoWise survey: cross-sectional study
Source: BMC Public Health. 2024 Jul 4;24:1781. doi: 10.1186/s12889-024-19293-0 (PMC11223381; doi:10.1186/s12889-024-19293-0)
Supplement: Supplementary file 1 — Additional file 1. [file 12889_2024_19293_MOESM1_ESM.docx]

| **Prevention Measure Use** | **Definition** |
| --- | --- |
| Long Sleeves/Pants | Wearing clothing that covers most of the body to reduce skin exposure and decrease the likelihood of mosquito bites |
| Insect Repellent | Applying substances (such as DEET) on exposed skin or clothing designed to deter mosquitoes and other insects from landing and biting |
| Electric Zapper | A device that attracts and kills flying insects through electrocution |
| Electric Fan | Using air circulation provided by a fan to deter mosquitoes, as they are weak fliers |
| Outlet Plug-in Repellent | An electronic device that emits substances, such as insecticides or natural oils, into the air to repel insects, plugged directly into an electrical outlet indoors |
| Window/Door Screens | Mesh barriers installed on windows and doors to prevent mosquitoes and other insects from entering indoor spaces while allowing air flow |
| Bedroom Windows Closed | Keeping windows in sleeping areas shut to prevent the entry of mosquitoes and other insects, especially during peak biting hours |
| Mosquito Bed Net | A net, usually made of fine mesh, placed over sleeping areas to protect individuals from mosquitoes and other insects while sleeping |
| Natural Methods | Using plant-based or other natural substances to repel mosquitoes, such as citronella, eucalyptus oil, garlic, or other herbal extracts |
| Removing Breeding Sites | Eliminating sources of standing water that facilitate mosquito breeding, such as clogged gutters, old tires, and containers that can hold water |
| Other | Any additional measures not listed here but used by individuals to prevent mosquito bites or reduce mosquito populations |
| None | No specific actions taken to prevent mosquito bites or control mosquito populations |

Additional file 1

Table defining prevention measures
