# Supplementary material for: Determinants of intended prevention behaviour against mosquitoes and mosquito-borne viruses in the Netherlands and Spain using the MosquitoWise survey: cross-sectional study
Source: BMC Public Health. 2024 Jul 4;24:1781. doi: 10.1186/s12889-024-19293-0 (PMC11223381; doi:10.1186/s12889-024-19293-0)
Supplement: Supplementary file 2 — Additional file 2. [file 12889_2024_19293_MOESM2_ESM.docx]

# **Additional file 2**

# **Measurement Invariance**

# **Methods**

Multi-group confirmatory factor analysis (MGCFA), a form of measurement invariance (MI), was performed to assess whether the survey can be used to compare perceptions of and behavior towards mosquito borne viruses (MBV’s) between the Netherlands and Spain. MGCFA assesses if a survey given to groups under different conditions will still measure the same outcomes; thus, groups (in this case NL and ES) can be compared directly. Three levels of measurement invariance tests were sequentially carried out using the WLS(MV) estimator: 1) Configural invariance (the items within each construct are the same across all countries), 2) Metric invariance (the constructs have the same meaning for all countries), and 3) Scalar invariance (the intercepts are the same for all countries so direct comparisons can be made).^1^ We examined invariance based on changes in CFI (∆CFI) and changes in RMSEA (∆RMSEA) between invariance levels as our primary tests of measurement invariance, where a ∆CFI of ≤ 0·010 along with a ∆ RMSEA of ≤ 0·015 indicate invariance.^2^ If any level of invariance failed, testing for partial invariance is completed by identifying items that could have an underlying effect.

## **Results**

The results of the measurement invariance test and the goodness of fit are shown in table 1. Configural and metric invariance was supported as shown by the ∆CFI of -0·005 and ∆RMSEA of 0·002 (table 1). The scalar invariance model was not supported, so question intercept values were checked, revealing that the question SUSmbv (I am worried about getting sick from a mosquito-borne virus in my country of residence) part of the perceived susceptibility construct, contributed to the fit difference between the Netherlands and Spain. Scalar partial invariance was tested not constraining SUSmbv. Based on the ∆CFI (0·01) and ∆RMSEA (0·005), scalar partial invariance showed a more acceptable fit in comparison to scalar invariance (table 1). Thus, SUSmbv was not included in the Susceptibility construct and the final health belief model (HBM) score.

**Table 1.** **Results of multiple levels of measurement invariance between the Dutch and Spanish responses.**

| **Level** | **χ2** | ***df*** | **CFI** | **RMSEA** | **RMSEA 95%** | **∆CFI** | **∆RMSEA** |
| --- | --- | --- | --- | --- | --- | --- | --- |
| **Configural** | 401·655 | 274 | 0·977 | 0·032 | 0·032-0·047 | - | - |
| **Metric** | 438·163 | 287 | 0·972 | 0·034 | 0·027-0·040 | 0·005 | 0·002 |
| **Scalar** | 645·428 | 300 | 0·937 | 0·050 | 0·045-0·054 | 0·035 | 0·016 |
| **Scalar partial^1^** | 509·672 | 299 | 0·962 | 0·039 | 0·033-0·044 | 0·010 | 0·005 |

1 Scalar partial without SUSmbv

## **References:**

1. Putnick DL, Bornstein MH. Measurement Invariance Conventions and Reporting: The State of the Art and Future Directions for Psychological Research. Dev Rev. 2016;41:71-90.

2. Chen FF. Sensitivity of Goodness of Fit Indexes to Lack of Measurement Invariance. Structural Equation Modeling: A Multidisciplinary Journal. 2007;14(3):464-504.
