# Supplementary material for: Determinants of intended prevention behaviour against mosquitoes and mosquito-borne viruses in the Netherlands and Spain using the MosquitoWise survey: cross-sectional study
Source: BMC Public Health. 2024 Jul 4;24:1781. doi: 10.1186/s12889-024-19293-0 (PMC11223381; doi:10.1186/s12889-024-19293-0)
Supplement: Supplementary file 3 — Additional file 3. [file 12889_2024_19293_MOESM3_ESM.docx]

Additional file 3

Table of Information preferences of survey participants in the Netherlands and Spain, 2022.

| **Information on Mosquitoes and Mosquito-Borne Viruses** | | | |
| --- | --- | --- | --- |
| **Characteristic** | **Netherlands** | **Spain** | **chi-squared test** |
|  | **N = 438** | **N = 475** |  |
| **Current Information Source** | **Count (Percent)** | | **p value** |
| Health professionals | 11 (2.51) | 67 (14.11) | <0.0001 |
| Government website | 22 (5.02) | 44 (9.26) | 0.01 |
| Social media | 54 (12.33) | 95 (20.00) | 0.002 |
| Family and friends | 23 (5.25) | 88 (18.53) | <0.0001 |
| Educational institutes | 11 (2.51) | 38 (8.00) | 0.0002 |
| Institutional websites | 12 (2.74) | 52 (10.95) | <0.0001 |
| Television and news channels | 64 (14.61) | 174 (36.63) | <0.0001 |
| Print newspapers | 25 (5.71) | 57 (12.00) | 0.001 |
| Radio | 15 (3.42) | 35 (7.37) | 0.009 |
| Communication campaign | 6 (1.37) | 40 (8.42) | <0.0001 |
| None of the above | 310 (70.78) | 186 (39.16) | <0.0001 |
| **Preferred Information Source** | **Count (Percent)** | | **p value** |
|  | **N = 370** | **N = 447** |  |
| Health professionals | 70 (18.92) | 271 (60.63) | <0.0001 |
| Government website | 189 (51.08) | 181 (40.49) | <0.0001 |
| Social media | 144 (38.92) | 145 (32.44) | <0.0001 |
| Family and friends | 23 (6.22) | 70 (15.66) | <0.0001 |
| Educational institutes | 44 (11.89) | 139 (31.10) | <0.0001 |
| Institutional websites | 72 (19.46) | 162 (36.24) | <0.0001 |
| Television and news channels | 189 (51.08) | 264 (59.06) | <0.0001 |
| Print newspapers | 71 (19.19) | 117 (26.17) | <0.0001 |
| Radio | 79 (21.35) | 104 (23.27) | <0.0001 |
| Communication campaign | 76 (20.54) | 191 (42.73) | <0.0001 |

*IQR* Interquartile Range. Percentages were calculated per row, using the total number of participants who indicated they received information or indicated they want to receive information. p values, for pairwise comparison between the Netherlands and Spain, were calculated using chi-square tests.
